# A new species of *Orthobula* Simon, 1897 (Araneae, Trachelidae) from South China

**DOI:** 10.3897/BDJ.10.e94202

**Published:** 2022-10-21

**Authors:** Mengzhen Zhang, Ning Ma, Zimin Jiang, Yonghong Xiao, Keke Liu

**Affiliations:** 1 College of Life Science, Jinggangshan University, Ji'an, China College of Life Science, Jinggangshan University Ji'an China

**Keywords:** Aranei, Jiangxi Province, taxonomy, trachelids

## Abstract

**Background:**

Only one trachelid species, *Trachelassinensis* Chen, Peng & Zhao, 1995 has been recorded from Jiangxi Province to date.

**New information:**

A new species, *Orthobulajiangxi* Liu, **sp. n.**, is described from Jiangxi Province of China, based on both sexes. Morphological illustrations are provided and its distribution is mapped.

## Introduction

The genus *Orthobula* Simon, 1897 is characterised by the large number of paired ventral spines on the tibiae and metatarsi, the presence of pits across the entire carapace surface, the posteriorly positioned spermathecae of the female epigyne and the swollen palpal tegulum and the finely coiled embolus on the male palp (Haddad et al. 2022). They usually live in leaf litter, woody debris, tree bark, under stones or on the forest floor. According to the World Spider Catalog (2022), there are 23 valid species recorded from the Asian (15 species), African (7 species) and South American (1 species) regions (World Spider Catalog 2022). More than 1/3 of these (8 species) were recorded from Chinese provinces, including Xizang, Beijing, Hunan, Shaanxi, Qinghai, Taiwan, Guangdong and Fujian (World Spider Catalog 2022). No species have been recorded from the other provinces of this huge country.

*Orthobula* has a turbulent taxonomic history over the last more than a century. This genus was placed in Liocraninae as part of Clubionidae by Simon (1897). Then it was transferred to the subfamily Phrurolithinae in Liocranidae by Deeleman-Reinhold (2001). Later, it was transferred to the Corinnidae by Bosselaers and Jocqué (2002). Twelve years later, Ramírez (2014) elevated Phrurolithidae to family status and the treated genus was placed in it. In 2016, Wheeler et al. (2016) transferred the genus to the Trachelidae, based on the molecular phylogenetic analysis. Morphological details, based on the sexual dimorphic characters of this genus, were not revealed until Haddad et al. (2022) published their work. Their works have greatly contributed to a better understanding of the group.

Recently, in Jiangxi Province, many spider taxa have been discovered, such as Agelenidae (Liu et al. 2020a, Liu et al. 2021), Dictynidae (Liu et al. 2018), Oonopidae (Liu et al. 2016, Liu et al. 2019), Phrurolithidae (Liu et al. 2020b, Liu et al. 2020c, Liu et al. 2021, Liu et al. 2022d), Salticidae (Liu et al. 2017b, Liu et al. 2022a), Thomisidae (Liu et al. 2017a, Liu et al. 2022c), Gnaphosidae (Liu et al. 2022b) and Trachelidae (this study). These discoveries support the fact that this Province is one of the biodiversity hotspots for China. When we collected ground spiders from this Province, an undescribed species was discovered and the present paper aims to provide detailed description of this new species.

## Materials and methods

Specimens were examined using a Zeiss Stereo Discovery V12 stereomicroscope with Zoom Microscope System. Further details were studied using a Zeiss Axio Scope A1 compound microscope with a KUY NICE CCD. Both the male palps and female epigyne were detached from the spider body and observed in 80−85% ethanol. The specimens were stored in 75% ethanol after photography. All specimens are deposited in the Animal Specimen Museum, College of Life Science, Jinggangshan University (ASM-JGSU).

All morphological measurements were taken using a stereomicroscope (AxioVision SE64 Rel. 4.8.3) and are given in millimetres. The body length of each specimen does not include the spinnerets. Leg measurements are given as total length (femur, patella, tibia, metatarsus, tarsus).

Terminology of the copulatory organs follows Ramírez (2014) and Haddad et al. (2022). Leg spination was documented by dividing each leg segment into two aspects, dorsal and ventral, the latter being divided into prolateral and retrolateral, for example, I tibia plv (prolateral ventral) 6, rlv (retrolateral ventral) 4. The abbreviations used in the figures are as follows: AER = anterior eye row; ALE = anterior lateral eye; AME = anterior median eye; Bu = bursa; CD = copulatory duct; CO = copulatory opening; Em = embolus; FD = fertilisation duct; IS = intercalary sclerite; MOA = median ocular area; PER = posterior eye row; PLE = posterior lateral eye; PME = posterior median eye; RTA = retrodital tibial apophysis; S1 = primary spermatheca; S2 = secondary spermatheca; SBB = sclerotised bursal base; St = subtegulum; Tu = tubercle; VFA = ventrodistal femoral apophysis.

## Taxon treatments

### Orthobula jiangxi

Liu sp. n.

48BDBCB5-9119-5351-810B-C1BCD9A2EF3F

Tra-10

EF27B5E4-34FD-4984-B732-93900E508626

#### Materials

- **Type status:** Holotype. **Occurrence:** recordedBy: Liu Ke-Ke; individualCount: 1; sex: male; lifeStage: adult; occurrenceID: 2F57207E-EB1E-58F4-B705-DC8F3574C909; **Taxon:** scientificName: Orthobulajiangxi Liu, sp. n.; **Location:** country: China; stateProvince: Jiangxi; locality: Yichun City, Wanzai County, Luocheng Town, Jiulongshan Forest Park, Zuojiashan Village; verbatimElevation: 164 m; verbatimCoordinates: 28°21'07.52"N, 114°30'27.58"E; georeferenceProtocol: GPS; **Event:** samplingProtocol: sieving; eventDate: 02/06/2021
- **Type status:** Paratype. **Occurrence:** recordedBy: Liu Ke-Ke; individualCount: 1; sex: female; lifeStage: adult; occurrenceID: 29043311-8D64-5D5E-BE6D-0CE0EB7D66A6; **Taxon:** scientificName: Orthobulajiangxi Liu, sp. n.; **Location:** country: China; stateProvince: Jiangxi; locality: Yichun City, Wanzai County, Luocheng Town, Jiulongshan Forest Park, Zuojiashan Village; verbatimElevation: 164 m; verbatimCoordinates: 28°21'07.52"N, 114°30'27.58"E; georeferenceProtocol: GPS; **Event:** samplingProtocol: sieving; eventDate: 02/06/2021
- **Type status:** Paratype. **Occurrence:** recordedBy: Liu Ke-Ke; individualCount: 1; sex: female; lifeStage: adult; occurrenceID: 548ED434-EB15-5A35-8DA2-CA057FF69A08; **Taxon:** scientificName: Orthobulajiangxi Liu, sp. n.; **Location:** country: China; stateProvince: Jiangxi; locality: Yichun City, Wanzai County, Luocheng Town, Jiulongshan Forest Park, Zuojiashan Village; verbatimElevation: 311 m; verbatimCoordinates: 28°22'50.24"N, 114°29'09.44"E; georeferenceProtocol: GPS; **Event:** samplingProtocol: sieving; eventDate: 02/06/2021
- **Type status:** Paratype. **Occurrence:** recordedBy: Liu Ke-Ke; individualCount: 1; sex: female; lifeStage: adult; occurrenceID: 0677951F-0AAF-50C4-B77C-68822624272E; **Taxon:** scientificName: Orthobulajiangxi Liu, sp. n.; **Location:** country: China; stateProvince: Jiangxi; locality: Ji’an City, Anfu County, Taishan Town, Yangshimu Scenic Spot, near Buffalo Grand Valley; verbatimElevation: 541 m; verbatimCoordinates: 27°31'39.69"N, 114°14'37.18"E; georeferenceProtocol: GPS; **Event:** samplingProtocol: sieving; eventDate: 05/04/2021
- **Type status:** Paratype. **Occurrence:** recordedBy: Liu Ke-Ke; individualCount: 3; sex: female; lifeStage: adult; occurrenceID: 6E50097E-0B38-5A20-B077-ABAFE94924B6; **Taxon:** scientificName: Orthobulajiangxi Liu, sp. n.; **Location:** country: China; stateProvince: Jiangxi; locality: Ganzhou City, Shangyou County, Wuzhifeng Town, Wuzhifeng drift; verbatimElevation: 451 m; verbatimCoordinates: 25°59'29.64"N, 114°10'51.30"E; georeferenceProtocol: GPS; **Event:** samplingProtocol: sieving; eventDate: 10/01/2020
- **Type status:** Paratype. **Occurrence:** recordedBy: Liu Ke-Ke; individualCount: 3; sex: male; lifeStage: adult; occurrenceID: 98379F85-AC1B-5B2B-BDDE-4845613C5AEB; **Taxon:** scientificName: Orthobulajiangxi Liu, sp. n.; **Location:** country: China; stateProvince: Jiangxi; locality: Ganzhou City, Shangyou County, Wuzhifeng Town, Wuzhifeng drift; verbatimElevation: 451 m; verbatimCoordinates: 25°59'29.64"N, 114°10'51.30"E; georeferenceProtocol: GPS; **Event:** samplingProtocol: sieving; eventDate: 10/01/2020
- **Type status:** Paratype. **Occurrence:** recordedBy: Liu Ke-Ke; individualCount: 1; sex: male; lifeStage: adult; occurrenceID: A2C4F0D0-950F-5089-9664-CF44690C5548; **Taxon:** scientificName: Orthobulajiangxi Liu, sp. n.; **Location:** country: China; stateProvince: Jiangxi; locality: Ganzhou City, Shangyou County, Wuzhifeng Town, Huangshakeng Village; verbatimElevation: 469 m; verbatimCoordinates: 25°59'43.70"N, 114°10'49.24"E; georeferenceProtocol: GPS; **Event:** samplingProtocol: sieving; eventDate: 10/01/2020
- **Type status:** Paratype. **Occurrence:** recordedBy: Liu Ke-Ke; individualCount: 1; sex: female; lifeStage: adult; occurrenceID: 7FAF4DA4-73DE-5F09-8881-0AEB75151EEE; **Taxon:** scientificName: Orthobulajiangxi Liu, sp. n.; **Location:** country: China; stateProvince: Jiangxi; locality: Ganzhou City, Shangyou County, Wuzhifeng Town, Huangshakeng Village; verbatimElevation: 469 m; verbatimCoordinates: 25°59'43.70"N, 114°10'49.24"E; georeferenceProtocol: GPS; **Event:** samplingProtocol: sieving; eventDate: 10/01/2020
- **Type status:** Paratype. **Occurrence:** recordedBy: Liu Ke-Ke; individualCount: 1; sex: female; lifeStage: adult; occurrenceID: 240ED723-2B30-5D3C-AC28-B6E4C2579102; **Taxon:** scientificName: Orthobulajiangxi Liu, sp. n.; **Location:** country: China; stateProvince: Jiangxi; locality: Ganzhou City, Longnan County, Jiulianshan Forest Farm, Gaofeng Entrance; verbatimElevation: 417 m; verbatimCoordinates: 24°37'12.53"N, 114°33'01.49"E; georeferenceProtocol: GPS; **Event:** samplingProtocol: sieving; eventDate: 10/06/2020
- **Type status:** Paratype. **Occurrence:** recordedBy: Liu Ke-Ke; individualCount: 2; sex: female; lifeStage: adult; occurrenceID: EE45D40B-5BA8-5846-B697-06369FC65AED; **Taxon:** scientificName: Orthobulajiangxi Liu, sp. n.; **Location:** country: China; stateProvince: Jiangxi; locality: Ganzhou City, Chongyi County, Reshui Town, Nanguotianshan Savannah Scenic Spot, near parking lot,; verbatimElevation: 833 m; verbatimCoordinates: 25°27'28.63"N, 113°55'22.42"E; georeferenceProtocol: GPS; **Event:** samplingProtocol: sieving; eventDate: 10/02/2020
- **Type status:** Paratype. **Occurrence:** recordedBy: Liu Ke-Ke; individualCount: 2; sex: female; lifeStage: adult; occurrenceID: F04132F5-CBE9-5BA2-B21C-D045D618A3FE; **Taxon:** scientificName: Orthobulajiangxi Liu, sp. n.; **Location:** country: China; stateProvince: Jiangxi; locality: Ji’an City, Qingyuan District, Jinggangshan University, in campus,; verbatimElevation: 87 m; verbatimCoordinates: 27°06'48.20"N, 115°01'29.01"E; georeferenceProtocol: GPS; **Event:** samplingProtocol: sieving; eventDate: 11/30/2013
- **Type status:** Paratype. **Occurrence:** recordedBy: Liu Ke-Ke; individualCount: 1; sex: male; lifeStage: adult; occurrenceID: D6B48B20-B669-5424-801B-3D052F7D5796; **Taxon:** scientificName: Orthobulajiangxi Liu, sp. n.; **Location:** country: China; stateProvince: Jiangxi; locality: Ji’an City, Jizhou District, Luling Zoology Park; verbatimElevation: 103 m; verbatimCoordinates: 27°08'41.40"N, 115°00'35.62"E; georeferenceProtocol: GPS; **Event:** samplingProtocol: sieving

#### Description

**Male** (holotype). Total length 2.05 mm.

**Figure 1.**
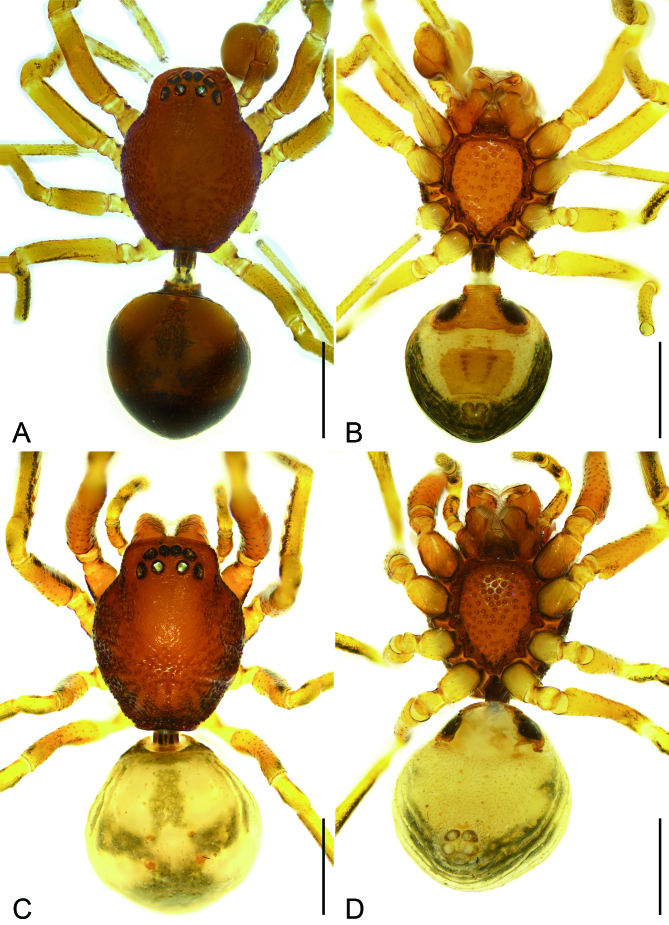
*Orthobulajiangxi* **Liu, sp. n.**, male holotype and female paratype (**A–D**). **A–B** Male habitus, dorsal and ventral view; **C–D** Female habitus, dorsal and ventral view. Scale bars: 0.5 mm.

Carapace (Fig. 1A and B) 0.94 mm long, 0.72 mm wide, anteriorly narrowed to 0.6 × its maximum width, with abundant large pore-bearing depressions on lateral and posterior parts. Eye sizes and interdistances: AER and PER procurved in dorsal view; AME 0.04, ALE 0.06, PME 0.05, PLE 0.05, AME−AME 0.02, AME−ALE 0.02, PME−PME 0.06, PME−PLE 0.05, AME−PME 0.04, AME−PLE 0.12, ALE−ALE 0.14, PLE−PLE 0.24, ALE−PLE 0.06; MOA 0.13 long, front width 0.1, back width 0.16. Chelicerae with two promarginal (distal larger) and three retromarginal teeth (median largest). Endites longer than wide, with sparse pore-bearing depressions. Labium wider than long, anteriorly with three pairs of strong setae, subposteriorly with a constriction, posteriorly with a row of pore-bearing depressions. Sternum strongly sclerotised, longer than wide, covered with many pits, anteromedially with a wide notch, laterally with sclerotised and thickened margin, posterior end blunt. Legs (Fig. 1A and B). Measurements: I 2.2 (0.62, 0.20, 0.62, 0.48, 0.28); II 1.87 (0.55, 0.22, 0.43, 0.42, 0.25); III 1.71 (0.49, 0.21, 0.31, 0.43, 0.27); IV 2.10 (0.56, 0.22, 0.45, 0.55, 0.32); leg formula 1423; spination: tibiae I plv 6, rlv 6 II plv 6, rlv 6; metatarsi I plv 4, rlv 4, II plv 4, rlv 4; tarsi I plv 3, rlv 3, II plv 3, rlv 3. Pedicel (Fig. 1A and B) 0.21 mm long, cylindrical, sclerotised. Abdomen (Fig. 1A, B) 0.91 mm long, 0.79 mm wide, scutum covering entire dorsum; venter with sclerotised epigastric region and trapezoidal inframamillary scutum in front of spinnerets.

Colouration (Fig. 1A and B). Carapace reddish-brown, with brown spots around depressions. Each eye with distinct black eye cup. Chelicerae and endites yellow. Endites reddish. Labium reddish, posterior part reddish-brown. Sternum reddish, with dark mottled sub-margin and reddish-brown margin. Legs yellow, with dark brown stripes on prolateral side of femorae. Pedicel dark brown. Abdomen reddish-brown, with three branched dark brown stripes anteriorly and large semi-oval dark brown mark posteriorly; venter yellow, with reddish sclerotised epigastric plate anteriorly and scutum posteriorly.

**Figure 2.**
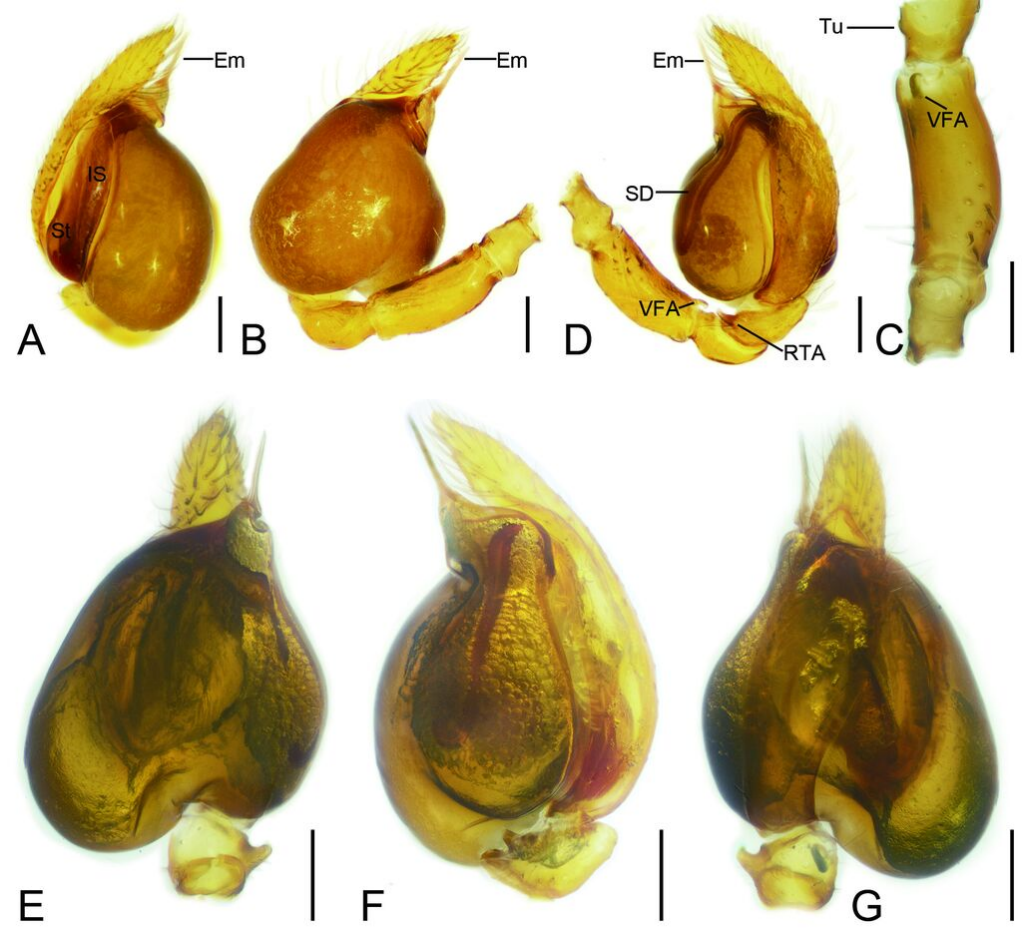
*Orthobulajiangxi* **Liu, sp. n.**, male palp of holotype (**A–G**). **A–B, D** Prolateral, prolater-ventral and retrolateral view; **C** detail of palpal femur, ventral view **E–G** ventral, retrolateral and dorso-retrolateral view. Abbreviations: Em = embolus; IS = intercalary sclerite; RTA = retrodistal tibial apophysis; St = subtegulum; Tu = tubercle; VFA = ventrodistal femoral apophysis. Scale bars: 0.1 mm.

Palp (Fig. 2). Palpal femur longer than patella + tibia, with small hook-shaped ventrodistal apophysis. Patella short, with small tubercle. Tibia slightly longer than patella, with finger-shaped retrodistal apophysis directed ventrally. Cymbium longer than femur + patella + tibia. Subtegulum lamellar, slightly sclerotised. Intercalary sclerite located between subtegulum and tegulum, lamellar. Tegulum swollen, slightly longer than wide, narrowing apically, with slight constriction in the middle part. Sperm duct narrow, n-shaped, with sharp turn in median part, nearly reaching the constriction. Embolus spine-like, short, reaching apex of cymbium.

**Figure 3.**
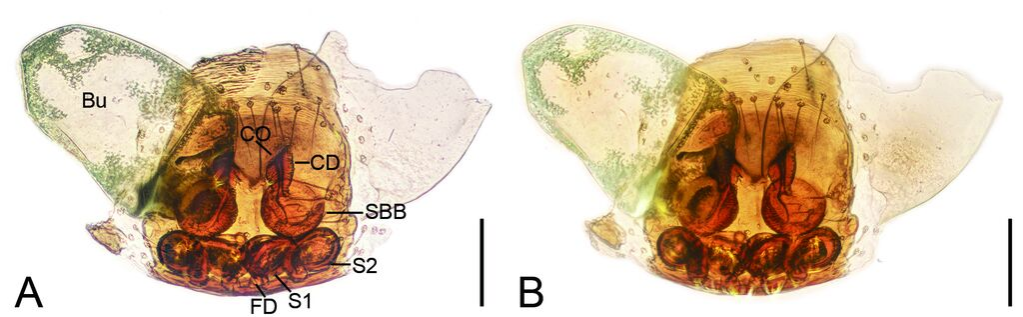
*Orthobulajiangxi* **Liu, sp. n.**, female epigyne of paratype (**A–B**). **A–B** ventral and dorsal (**B**) view. Abbreviations: Bu = bursa; CD = copulatory duct; CO = copulatory opening; FD = fertilisation duct; S1= primary spermatheca; S2 = secondary spermatheca, SBB = sclerotised bursal base. Scale bars: 0.1 mm.

**Female** (Fig. 1C and D and Fig. 3). As in male, except as noted. Total length 2.15 mm.

Carapace 1.03 mm long, 0.81 mm wide. Eye sizes and interdistances: AME 0.05, ALE 0.06, PME 0.07, PLE 0.07, AME−AME 0.01, AME−ALE 0.01, PME−PME 0.07, PME−PLE 0.02, AME−PME 0.04, AME−PLE 0.11, ALE−ALE 0.14, PLE−PLE 0.25, ALE−PLE 0.04. MOA 0.17 long, front width 0.12, back width 0.19. Pedicel 0.07 long. Abdomen (Fig. 1C and D) 0.98 long, 0.97 wide, without dorsal and ventral scutum. Leg measurements: I 2.26 (0.70, 0.24, 0.72, 0.57, 0.27); II 2.04 (0.61, 0.24, 0.45, 0.47, 0.27); III 1.98 (0.56, 0.22, 0.47, 0.43, 0.30); IV 2.62 (0.67, 0.25, 0.63, 0.71, 0.36).

Colouration as in Fig. 1C and D. Abdomen cream with black markings. The colour clearly differs from that of the male.

Epigyne (Fig. 3). Copulatory openings located at the posterior part of epigynal ridge, inclined, directed anteromedially. Copulatory ducts very short, shorter than width of sclerotised bursal base. Sclerotised bursal base round, separated by half their width. Bursae fan-shaped, very large, covering more than 2/3 of epigynal field. Sclerotised bursal base connected to oval secondary spermathecae by short ducts, longer than width of primary spermathecae. Secondary spermathecae connecting with oval primary spermathecae, closely touching each other, arranged in a line. Fertilisation ducts located at primary spermathecae, curved anteriorly.

#### Diagnosis

The male of this new species is similar to that of *Orthobulaspiniformis* Tso, Zhu, Zhang & Zhang, 2005 (Tso et al. 2005: 47, figs. 5 and 6) in having a spine-like and straight embolus, but can be distinguished from it by the hook-shaped ventrodistal femoral apophysis (vs. short and spine-like) and thin retrolateral tibial apophysis (vs. thick) (Fig. 2). The female of the new species resembles those of *O.aethiopica* Haddad, Jin & Platnick, 2022 (Haddad et al. 2022: 361, figs. 54 and 55), *O.arca* Haddad, Jin & Platnick, 2022 (Haddad et al. 2022: 364, figs. 59 and 60) and *O.spiniformis* (Tso et al. 2005: 47, figs. 5 and 6) in having round, slightly separated, sclerotised bursal base, but it can be easily distinguished by two pairs of spermathecae (vs. one) (Fig. 3).

#### Etymology

The species name is derived from the name of the type locality; noun in apposition.

#### Distribution

**Figure 4.**
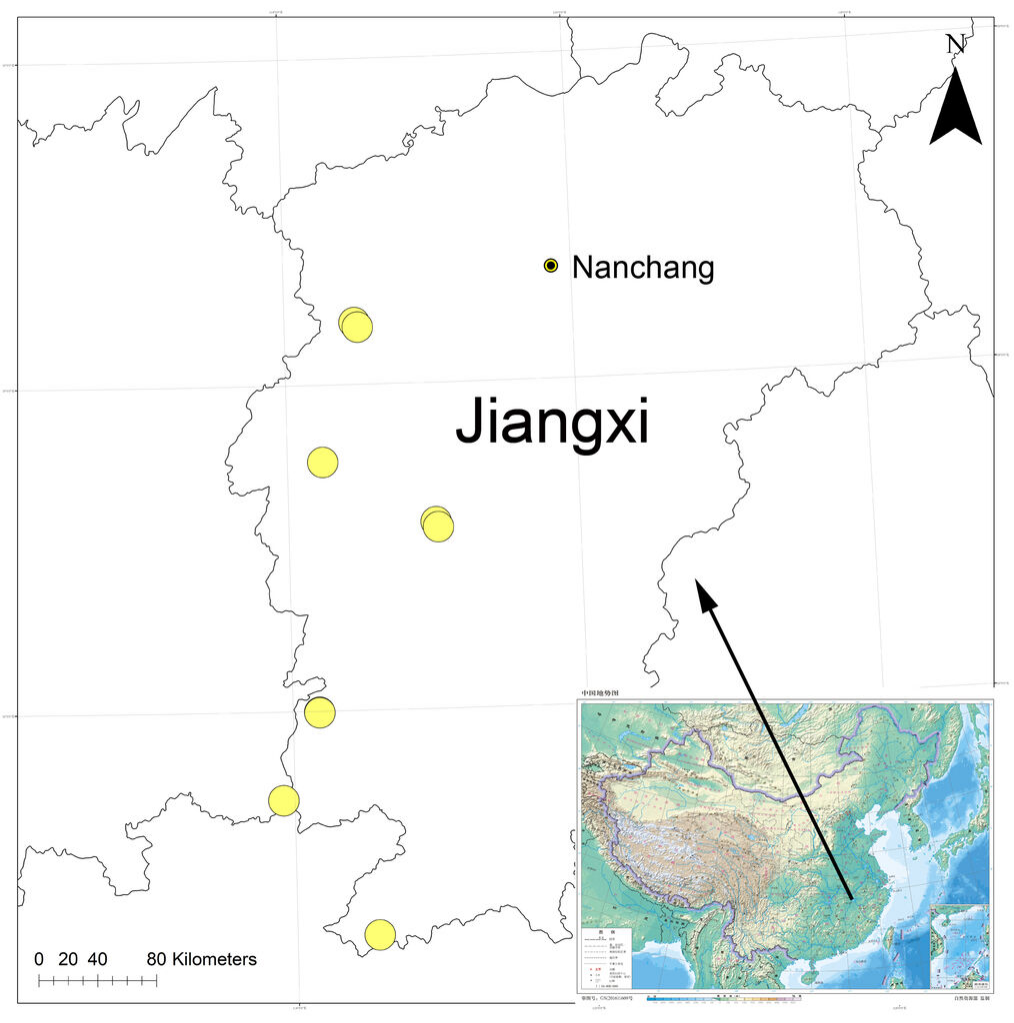
Records of *Orthobulajiangxi* **Liu, sp. n.**, from Jiangxi Province, China.

Known from Yichun, Ji’an and Ganzhou Cities in Jiangxi Province, China (Fig. 4). It seems that this species is more widespread within this Province.

#### Biology

It was collected from leaf litter in areas of broad-leaved forests in hilly areas.

#### Taxon discussion

The genus *Orthobula* has a wide distribution, with a rich population in forest litter in tropical to subtropical regions. However, this group has not received much attention in China and, until now, only eight known species have been reported from this huge country (World Spider Catalog 2022). The main reasons include the following: firstly, most species of *Orthobula* are difficult to collect and observe due to their very small body size; secondly, many *Orthobula* species are difficult to distinguish from their closely-related species, especially in males; finally, the descriptions of the new species from China were superficial and only a few ink drawings were provided, resulting in some difficulties for later taxonomic works.

## Supplementary Material

XML Treatment for Orthobula jiangxi
